# Global analysis of the haematopoietic and endothelial transcriptome during zebrafish development

**DOI:** 10.1016/j.mod.2012.10.002

**Published:** 2013-02

**Authors:** J.E. Cannon, E.S. Place, A.M.J. Eve, C.R. Bradshaw, A. Sesay, N.W. Morrell, J.C. Smith

**Affiliations:** a Department of Medicine, University of Cambridge, Cambridge CB2 0QQ, UK; b Wellcome Trust/Cancer Research UK Gurdon Institute, The Henry Wellcome Building of Cancer and Developmental Biology, University of Cambridge, Tennis Court Road, Cambridge CB2 1QN, UK; c Department of Zoology, University of Cambridge, Downing Street, Cambridge CB2 3EJ, UK; d Division of Systems Biology, MRC National Institute for Medical Research, The Ridgeway, Mill Hill, London NW7 1AA, UK

**Keywords:** Zebrafish, Haematopoiesis, Endothelial cell, High-throughput sequencing

## Abstract

In this paper, we use zebrafish embryos to characterise the transcriptome of the developing blood and endothelium, two cell types that are closely associated during development. High-throughput sequencing identified 754 genes whose transcripts are enriched threefold or more in blood and/or vascular endothelial cells compared with the rest of the embryo at 26–28 h post fertilisation. Of these genes, 388 were classified as novel to these cell types after cross-reference with PubMed and the zebrafish information network (ZFIN). Analysis by quantitative PCR and *in situ* hybridisation showed that 83% (*n* = 41) of these novel genes are expressed in blood or vascular endothelium. Of 10 novel genes selected for knockdown by antisense morpholino oligonucleotides, we confirmed that two, *tmem88a* and *trim2a*, are required for primitive erythropoiesis and myelopoiesis. Our results provide a catalogue of genes whose expression is enriched in the developing blood and endothelium in zebrafish, many of which will be required for the development of those cell types, both in fish and in mammals.

## Highlights

► Transcriptome of developing blood and vascular endothelial cells in zebrafish is described. ► 388 Novel genes expressed by blood and endothelial cells are identified. *► tmem88a* and *trim2a* are novel genes required for primitive erythropoiesis and myelopoiesis.

## 1 Introduction

Zebrafish are widely used in studies investigating haematopoietic and vascular development. They have several advantages over other vertebrate model systems, including access to hundreds of externally fertilised, transparent embryos that allow the visualisation of developmental processes *in vivo*. There are also a number of haematopoietic and vascular mutants previously found in large scale ENU mutagenesis screens (reviewed by Baldessari and Mione, 2008), and transgenic lines are available including the Tg(*fli1a:egfp*)^y1^ line used in this study (Baldessari and Mione, 2008; Lawson and Weinstein, 2002). For genes where mutants are not available, antisense morpholino oligonucleotides (morpholinos) can be used to knock down genes of interest. Finally, and importantly, there is a high degree of conservation of genes known to be important for vascular and haematopoietic development between zebrafish and higher organisms (Jing and Zon, 2011).

During early vertebrate embryo development blood and endothelial cells are found closely associated. In mammals they are initially found in the blood islands of the extra-embryonic yolk sac (Park et al., 2005), while during segmentation in zebrafish they are found intra-embryonically in the intermediate cell mass (ICM) of the ventral mesoderm (Detrich et al., 1995). In view of this close relationship it has been suggested that blood and endothelial cells have a common precursor cell, the haemangioblast (Sabin, 1920). Although there has been evidence to support this hypothesis from *in vitro* studies, it has only recently been shown that the haemangioblast exists *in vivo* (Park et al., 2005; Vogeli et al., 2006).

The factors controlling haemangioblast formation and the development of angioblasts (vascular endothelial cell precursors) and haematopoietic stem cells are incompletely understood. Several transcription factors are important for the formation of the haemangioblast. Stem cell leukaemia (*scl*, also known as *tal1*) null mice die *in utero* due to the complete absence of blood (Shivdasani et al., 1995). In zebrafish, morpholino knockdown of *scl* phenocopies the null mouse, but these embryos also have impaired vascular gene expression in the dorsal aorta and loss of intersegmental vessel (ISV) formation (Patterson et al., 2005). The Ets-1 related protein (*etsrp*, also known as *etv2*) was identified in a screen for novel genes affected in the *cloche* mutant, that lacks both blood and endothelial cells (Sumanas et al., 2005). Morpholino knockdown of *etsrp* leads to impaired vasculogenesis and myelopoiesis (Sumanas et al., 2008; Sumanas and Lin, 2006). *Fli1*, like *etsrp*, is an ETS transcription factor that is also important for haemangioblast formation. It has been suggested to act at the top of a transcriptional network driving blood and endothelial development by regulating other genes required for haemangioblast formation including *scl* and *etsrp* (Liu et al., 2008). The VEGF signalling pathway is also critical for vascular development. Loss of *Vegf* or its receptor *Flk1* in mice leads to death *in utero* due to failure to form the vasculature (Carmeliet et al., 1996; Shalaby et al., 1995). For erythrocyte development *Gata1* is a master regulator. *Gata1−/−* mice die *in utero* due to the failure of pro-erythrocytes to differentiate into mature erythrocytes (Fujiwara et al., 1996).

The identification of genes involved in blood and endothelial development is of significant therapeutic interest. We therefore sought to determine the transcriptome of developing haematopoietic and vascular endothelial cells *in vivo*. Previous studies have attempted to answer this question using microarrays (Covassin et al., 2006; Kalén et al., 2009; Sumanas et al., 2005; Wallgard et al., 2008; Weber et al., 2005; Wong et al., 2009). Here, because *fli1* is one of the earliest factors involved in haemangioblast formation, we have used a fluorescence-activated cell sorting (FACS) technique (Covassin et al., 2006) to isolate *gfp* positive (*gfp*+) and negative (*gfp*−) cells from transgenic Tg(*fli1a:egfp*)^y1^ embryos prior to high-throughput sequencing. This transgenic line utilises the *fli1a* promoter to drive *gfp* expression in blood and vascular endothelial cells, pharyngeal arch and neural crest derivatives (Lawson and Weinstein, 2002). Using this technique we have identified 388 novel genes expressed in the enriched population of blood and endothelial cells. Using morpholino knockdown we confirm that two of the genes identified, *tmem88a* and *trim2a*, are novel genes required for erythropoiesis and myelopoiesis in zebrafish.

## 2 Results

### 2.1 Isolation of vascular and haematopoietic cells from whole embryos

To identify genes involved in the development of endothelial and blood cells, we isolated *gfp*+ cells from dissociated 26–28 hpf Tg(*fli1a:egfp)*^y1^ transgenic zebrafish, where the *fli1a* promoter drives *gfp* expression in endothelial and haematopoietic cells and pharyngeal arch tissue (Lawson and Weinstein, 2002). This time-point was chosen because the intersegmental vessels are forming by angiogenesis and the haematopoietic stem cells are starting to arise from the ventral floor of the aorta (Bertrand et al., 2010; Isogai et al., 2003).

**Supplementary Fig. 1:**
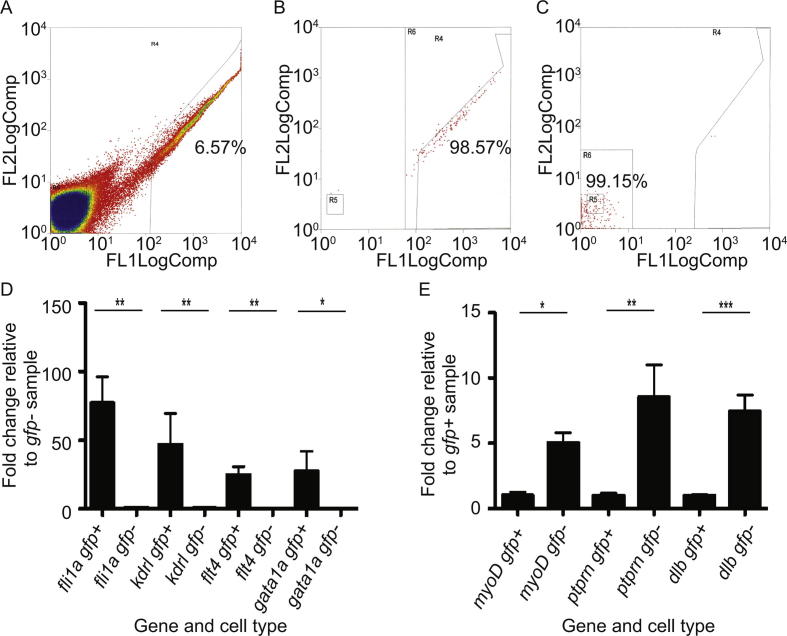
Isolation of pure populations of *gfp+* and *gfp−* cells from dissociated Tg(*fli1a:egfp*)^y1^ embryos. (A) Summary of FACS sort. *gfp+* cells were in R4 and *gfp-* cells in R5. Approximately 6.6% of cells were *gfp+*. Re-analysis of sorted cells demonstrated that 98.6% and 99.8% were true positive (B) or true negative respectively (C). Summary of relative expression of genes expected to be enriched in either *gfp+* (D) or *gfp−* (E) cells by qRT-PCR. Expression levels were normalised to *ef1α* prior to expressing fold changes relative to the *gfp−* (D) or *gfp+* (E) values. Error bars indicate SD. *n* = 3.

Approximately 6.6% of cells in 26–28 hpf Tg(*fli1a:egfp)*^y1^ embryos were *gfp+* by FACS (Supplementary Fig. 1A). A small proportion of cells from each sorted group were re-sorted to determine the purity of the cell populations. The *gfp+* population was always greater than 95% pure (Supplementary Fig. 1B) and the *gfp−* population greater than 99% (Supplementary Fig. 1C). As further validation for the purity of each population, qRT-PCR was performed on cDNA made from RNA isolated from the sorted cells. Genes expected to be enriched in the *gfp+* cells were indeed highly enriched. These included *fli1a* (77.2 ± 20.0-fold), *kdrl* (46.9 ± 23.8-fold), *flt4* (24.9 ± 6.2-fold) and *gata1a* (27.9 ± 14.0) (Supplementary Fig. 1D). Conversely, several genes not known to be highly expressed in vascular or haematopoietic cells (ZFIN, www.zfin.org) were more highly expressed in *gfp-* cells. These included *myoD* (5.1 ± 0.8-fold), *ptprn* (8.5 ± 2.5-fold), and *dlb* (7.4 ± 1.2-fold) (Supplementary Fig. 1E). Together these results indicate that we can isolate highly purified populations of *gfp+* and *gfp−* cells from Tg(*fli1a:egfp)*^y1^ zebrafish embryos.

### 2.2 Global analysis of genes enriched in *gfp* positive cells by massively parallel sequencing

**Supplementary Fig. 2:**
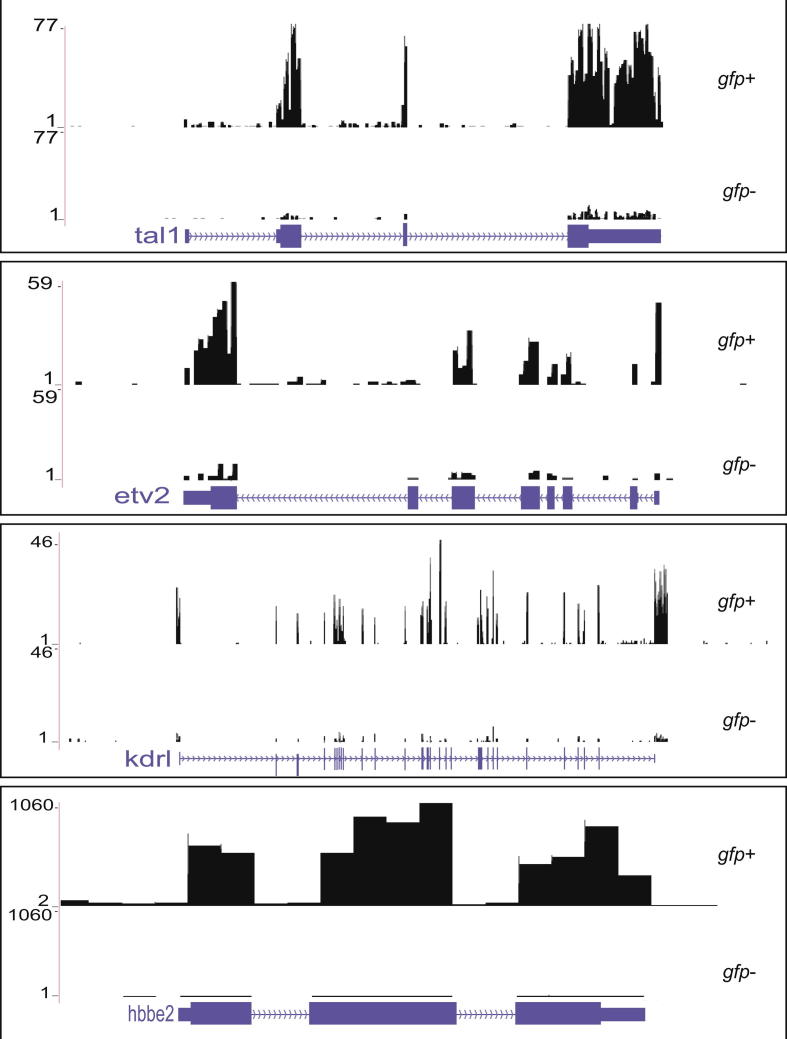
RNA-Seq read alignment to Zv8 zebrafish genome on UCSC genome browser. Illustrative alignments of reads from the first biological replicate of *gfp+* and *gfp−* libraries. The genes (RefSeq data) are found at the bottom of each box and consist of the gene name, intron (purple line containing arrows showing the direction of the gene), UTR (thin block) and exons (thicker block). The scale for the number of reads for each gene are on the left side.

**Fig. 1:**
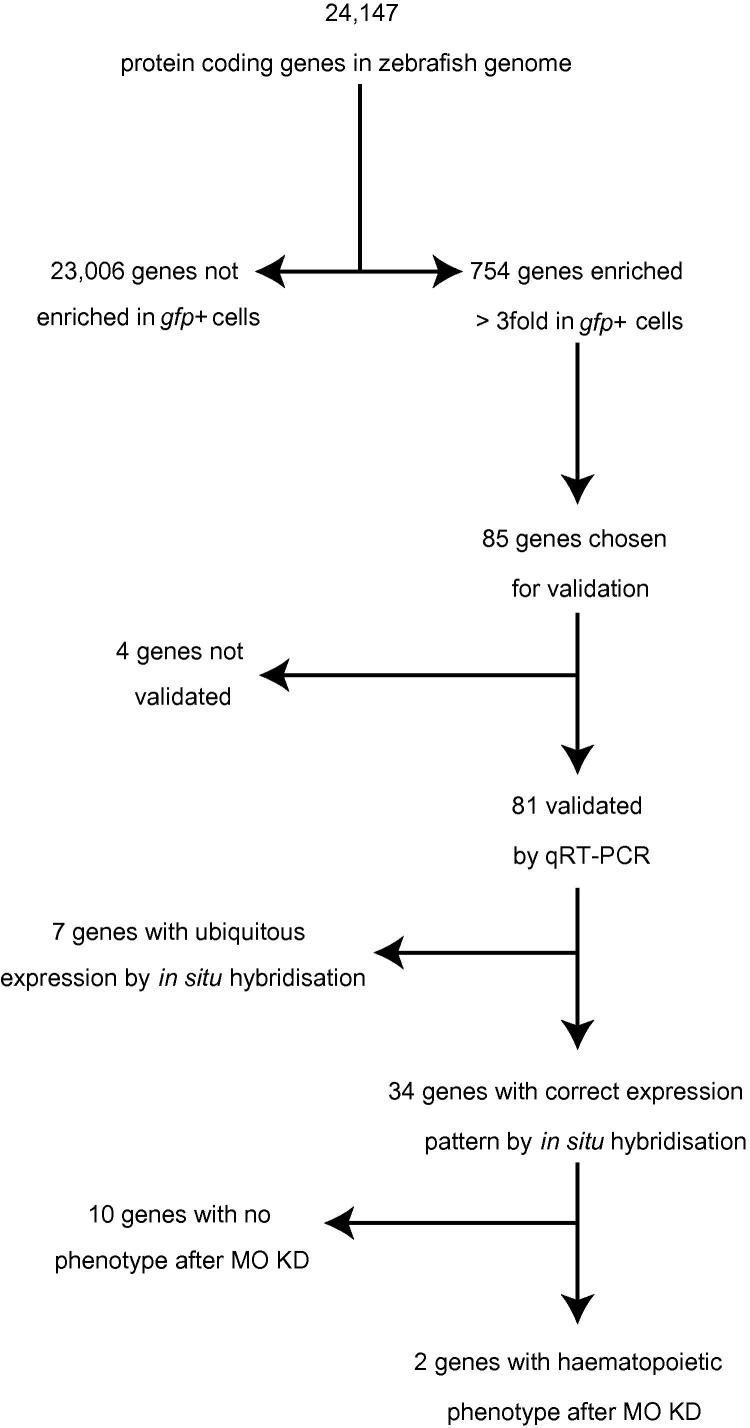
Overview detailing the analysis and validation of the high-throughput sequencing data. MO = morpholino, KD = knockdown.

The transcriptome of developing zebrafish blood and vascular endothelial cells was defined by undertaking high-throughput sequencing of cDNA made from sorted *gfp+* and *gfp*− cell populations derived from about 3000 Tg(*fli1a:egfp*)^y1^ embryos. We found that 754 protein-coding genes were enriched threefold or greater in the *gfp+* compared to the *gfp−* population of cells in both biological replicates (Fig. 1 and Supplementary Table 1). This group includes genes expected to be enriched such as *scl*, *etsrp*, *fli1a*, *gata1a*, haemoglobins and vegf receptors (Supplementary Fig. 2 and Table 1). Some genes known to be important for vascular development and/or haematopoiesis (like *ephrinb2*, *ephB4*, *jag2*, *notch1*, *notch3* and *unc5b*) were not enriched in the *gfp+* libraries (Adams et al., 1999; Hadland et al., 2004; Krebs et al., 2000; Lawson et al., 2002; Lu et al., 2004; Van de Walle et al., 2011; Wang et al., 1998). This is because these genes are also strongly expressed in other tissues including neural tissues (ZFIN).

**Supplementary Fig. 3:**
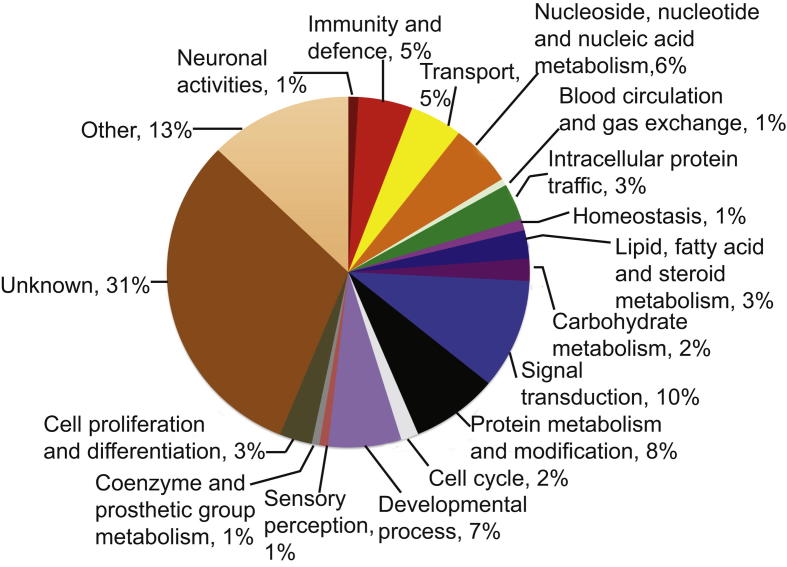
The biological functions of the 754 enriched genes using the Panther Ontology.

To identify the biological functions of the 754 genes we used the Panther classification system (Thomas et al., 2003) Genes involved in blood circulation and gas exchange (2.77-fold, *p* = 5.14E^−04^), immunity and defence (1.45-fold, *p* = 5.27E−05), transport (1.42-fold, *p* = 1.91E−04) and intracellular protein traffic (1.4-fold, *p* = 1.82E−03) were found to be the most significantly enriched compared to the whole zebrafish genome whereas neuronal activities (−2.28-fold, *p* = 8.57E−06), nucleoside, nucleotide and nucleic acid metabolism (−1.31-fold, *p* = 2.42E−04) and sensory perception (−1.59-fold, *p* = 0.024) were significantly under-represented (Table 1). One third of the genes, however, had an unclassified biological function (Supplementary Fig. 3). Use of ZFIN and PubMed revealed that 43% of the genes were already known to be expressed in either blood or endothelial cells in zebrafish, *Xenopus*, mouse, chick or humans, 6% in other tissues and organs like pharyngeal arch, pronephric duct, neural crest or heart, while 51% had an unknown expression pattern (Supplementary Table 1).

### 2.3 Validation of massively parallel sequencing genes

**Supplementary Fig. 4:**
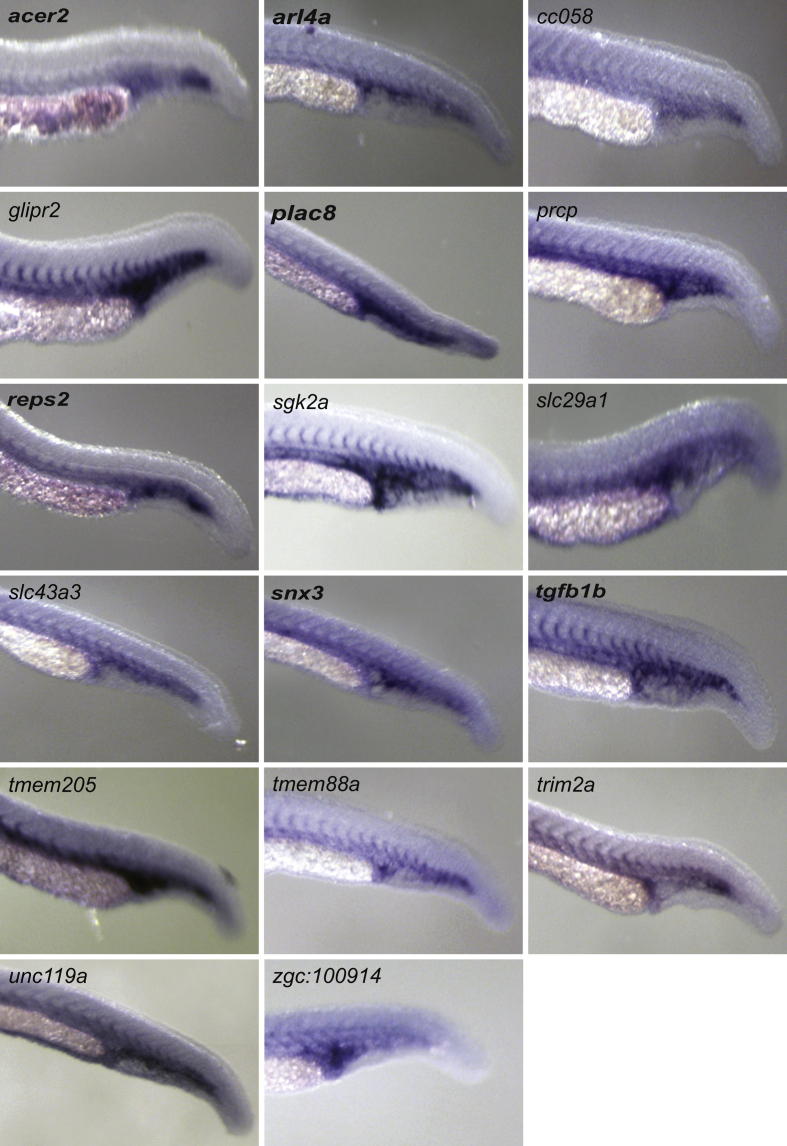
High powered tail view of embryos with blood and vascular cell *in situ* hybridisation expression patterns in 24–28 hpf embryos. All embryos are lateral views with anterior to left.

**Fig. 2:**
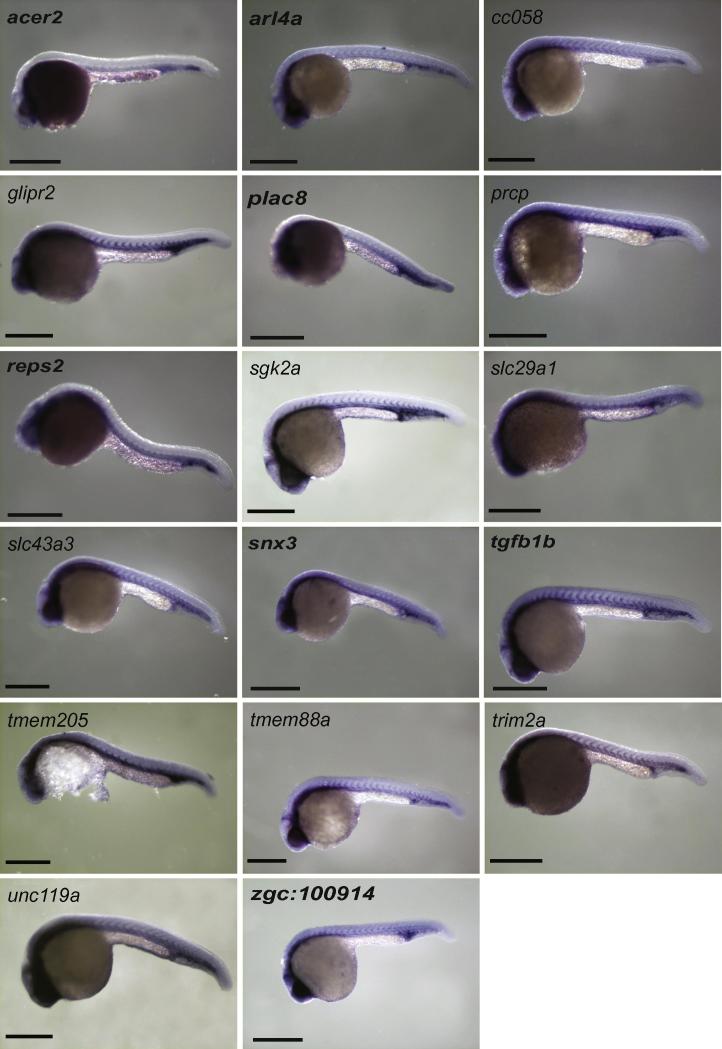
Genes with blood and vascular *in situ* hybridisation expression pattern in 24–28hpf embryos. All embryos are lateral views with anterior to left. Scale bars indicate 500 μm. High powered views of tail region of these embryos are shown in Supplementary Fig. 4.

**Fig. 3:**
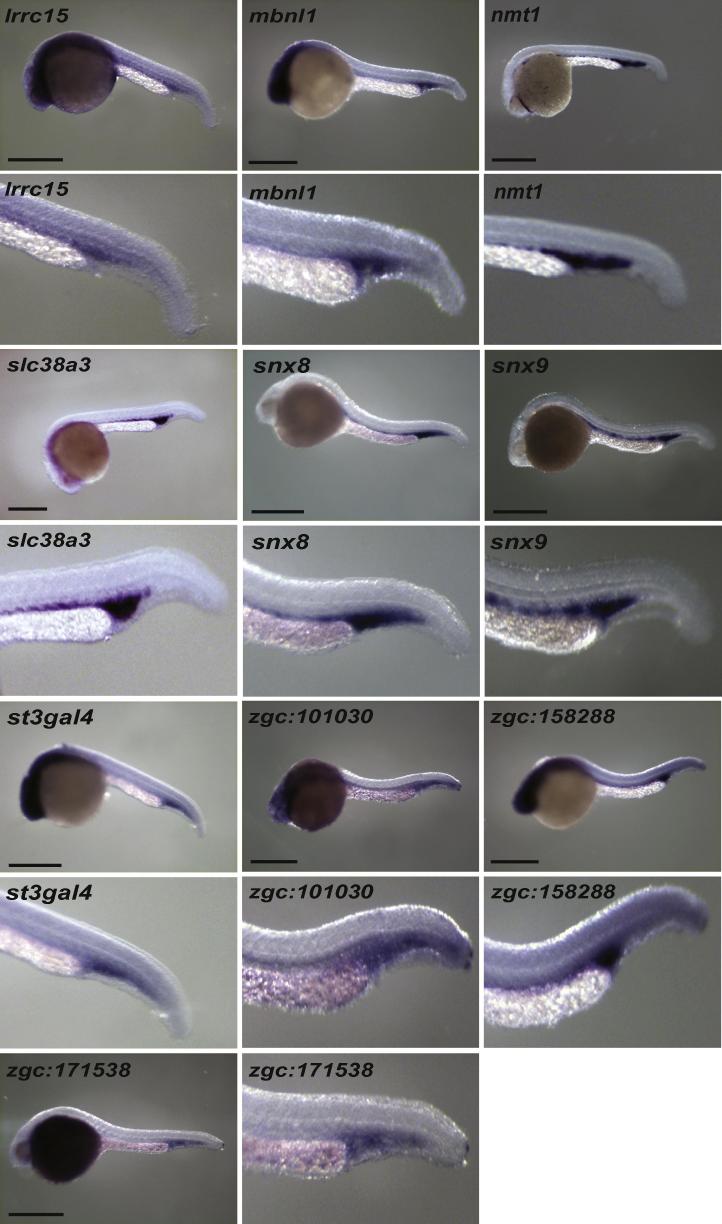
Genes with blood *in situ* hybridisation expression pattern in 24–28hpf embryos. All embryos are lateral views with anterior to left. Scale bars indicate 500 μm.

**Fig. 4:**
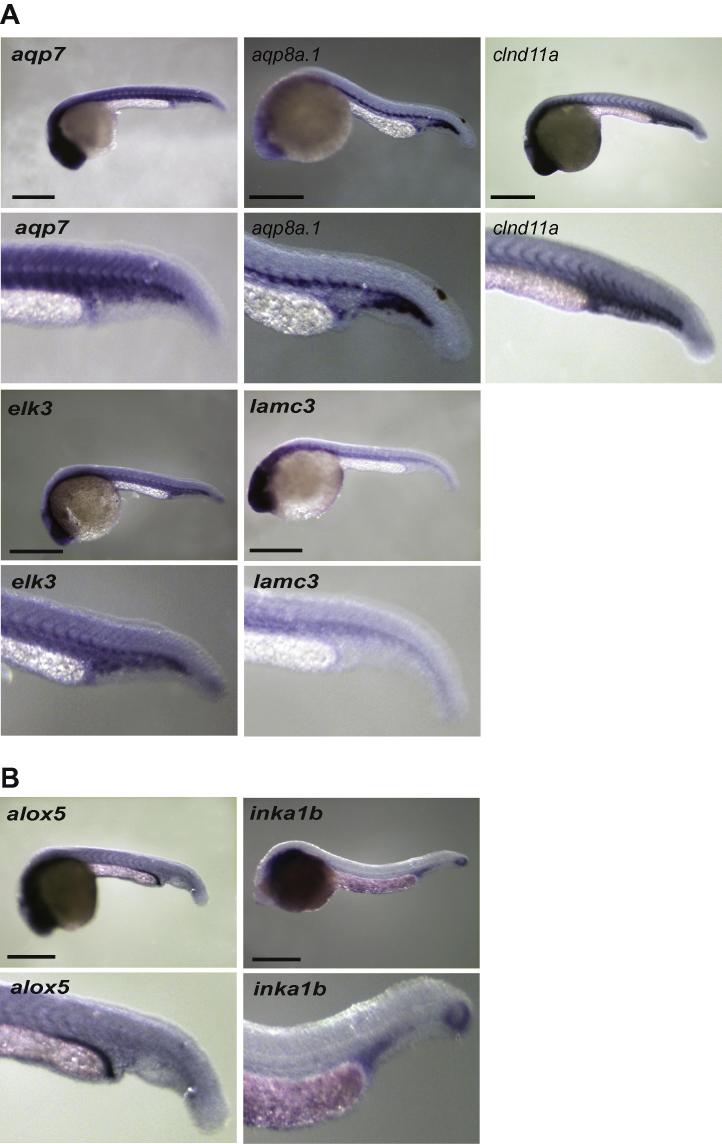
Remaining *in situ* hybridisation expression patterns in 24–28hpf embryos. (A) Vascular expression (B) Other. All embryos are lateral views with anterior to left. Scale bars indicate 500 μm.

Eighty-five genes without a known role in blood or endothelial cell development or angiogenesis were chosen for validation. Using the remaining total RNA that was used to make the initial libraries, eighty-one of the eighty-five genes could be validated by qRT-PCR (Table 2 and Supplementary Table 4). Because *gfp* expression in Tg(*fli1a:egfp*)^y1^ zebrafish is found in pharyngeal arch tissue and neural crest derivatives as well as in blood and endothelial cells (Lawson and Weinstein, 2002), we also selected forty-one genes to screen by whole mount *in situ* hybridisation. Of these, seventeen had restricted expression in both vascular endothelial and blood cells (Fig. 2 and Supplementary Fig. 4), 10 just in blood (Fig. 3), five in endothelial cells alone, one in pronephric duct and one in pharyngeal arch, endothelial cells and tailbud (Fig. 4 and Supplementary Table 2). The remaining seven genes had widespread expression (data not shown).

### 2.4 *Tmem88a* and *trim2a* morphants have reduced erythrocyte and myeloid cell formation

**Supplementary Fig. 5:**
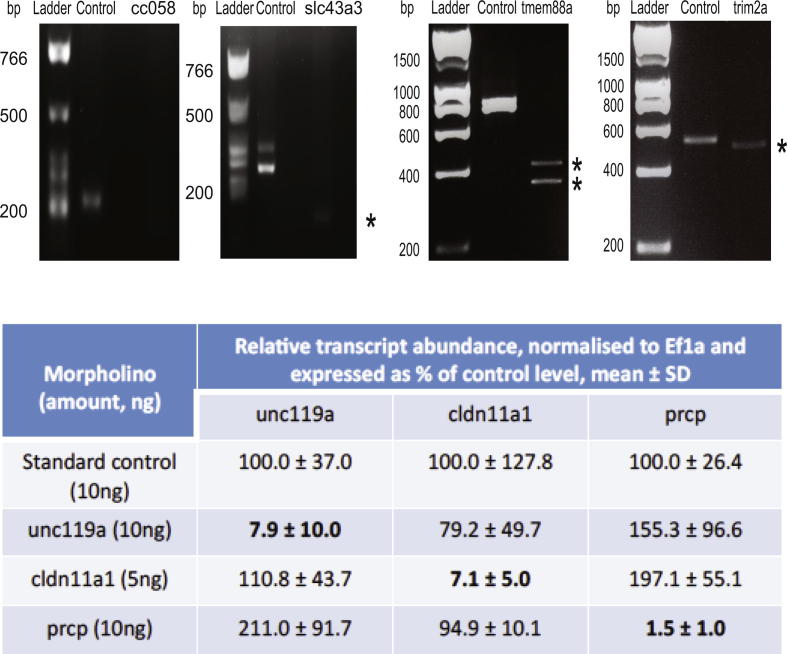
Confirmation of morpholino efficiency. Top panel shows splicing morpholinos designed to cause exon deletion detectable by RT-PCR. Bottom panel shows qPCR results for genes where morpholinos are designed to cause intron inclusion.

**Supplementary Fig. 6:**
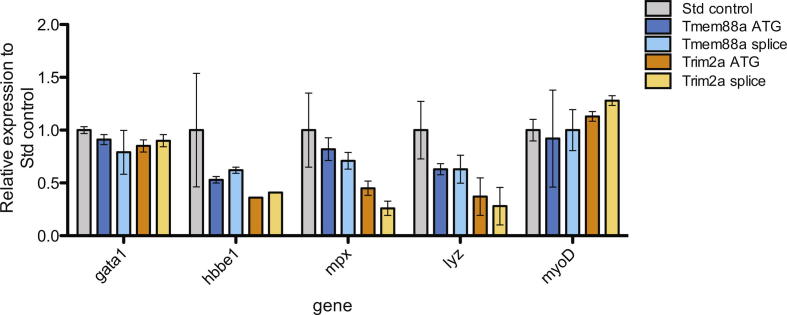
Quantitative PCR for selected genes in *tmem88a* and *trim2a* morphants. Graphs show relative expression compared to standard control injected embryos. *hbbe1* – haemoglobin beta embryonic 1, *mpx* – myelperoxidase, *lyz* – lysozyme, *myoD* – myogenic differentiation.

**Fig. 5:**
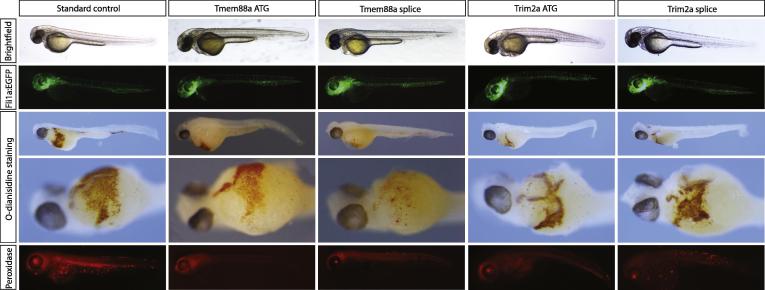
*Tmem88a* and *trim2a* morphants have reduced erythrocytes and myeloid cells as shown by O-dianisidine and peroxidase staining respectively at 48 h post fertilisation. In control embryos erythrocytes are present in axial vessels and returning to the heart across the yolk. Myeloid cells are found across the yolk and randomly around the remaining parts of the embryos. There is loss of erythrocytes and myeloid cells in both *trim2a* and *tmem88a* morphants (translation and splice blocking morpholinos) without any defect in vascular development. Panel row 1 shows representative brightfield images, row 2 epifluorescent images of Tg(*fli1a:*egfp) embryos, rows 3 and 4 show embryos post O-dianisidine staining and row 5 show embryos post peroxidase staining. All images are lateral views with anterior to left with the exception of row 4 which are ventral views.

Finally, as confirmation of the usefulness of this approach to identify genes required for the development of blood or endothelial cells, we inhibited gene function using antisense morpholino oligonucleotides. Ten genes with strong localised expression by *in situ* hybridisation were selected for study (Table 2). Loss of function of eight of these genes had no morphological effect and they had no vascular or blood phenotype when examined at 24 and 48 hfp despite gene knockdown (data not shown and Supplementary Fig. 5). Embryos lacking *tmem88a* or *trim2a* had normal vascular patterning at 24 and 48 hpf but reduced numbers of erythocytes and myeloid cells as judged by O-dianisidine and peroxidase staining respectively at 48 hpf (Fig. 5). For each gene this phenotype was observed with a translation-blocking and a splice-blocking morpholino. Surprisingly there was normal expression of *gata1a* at 24 hpf in the *tmem88a* and *trim2a* morphants but reduced expression of embryonic haemoglobin *hbbe1* along with myeloid markers *mpx* and *lyz* using qPCR (Supplementary Fig. 6). This suggests that the effect on erythrocyte development by these genes is downstream of *gata1*. The identification of 2 novel genes involved in primitive blood cell development confirms the validity of our approach used.

## 3 Discussion

Our experiments have identified 754 protein-coding genes that are enriched at least threefold in the *gfp+* population of Tg(*fli1a:egfp*)^y1^ zebrafish. The increased sensitivity of high-throughput sequencing is emphasised by the fact that a previous study using microarray technology and a twofold enrichment criterion identified only one-quarter the number of genes enriched in this study (Covassin et al., 2006). Of the 754 protein-coding genes enriched threefold in the *gfp+* population of cells, 43% were already known to be expressed in blood or endothelial cells by cross-referencing the genes with ZFIN and PubMed. A number of previous studies have been performed to isolate and characterise blood and/or vascular endothelial cells (Covassin et al., 2006; Kalén et al., 2009; Sumanas et al., 2005; Wallgard et al., 2008; Weber et al., 2005; Wong et al., 2009). The data from the current study complements these and provides a catalogue of the transcriptome of developing blood and vascular endothelial cells. Some genes expressed in blood or vascular endothelial cells, such *ephrinb2*, *ephB4*, *jag2*, *notch1*, *notch3* and *unc5b* (Adams et al., 1999; Hadland et al., 2004; Krebs et al., 2000; Lawson et al., 2002; Lu et al., 2004; Van de Walle et al., 2011; Wang et al., 1998) were not enriched in our study, possibly because they are also highly expressed in other tissues, particularly neural tissues.

Eighty-five genes without a known role in the development of blood or vascular endothelial cells or angiogenesis were chosen for validation by qRT-PCR. Eighty-one (95%) of these were validated in both biological replicates. As a key regulator of the transcriptional network driving blood and endothelial development, *fli1a* would be expected to be a good marker for isolating a pure population of blood and endothelial cells but it is also expressed in pharyngeal arch and neural crest derivatives. Our experiments therefore yield an enriched population of blood and endothelial cells rather than a population that is pure. (Lawson and Weinstein, 2002; Liu and Patient, 2008). In view of this, the expression pattern of forty-one of these novel genes were determined by whole mount *in situ* hybridisation. Thirty-four (83%) had a restricted expression in blood and/ or endothelial cells thus confirming the strength of our approach for identifying novel blood and vascular endothelial genes. Finally ten of these genes were then knocked down using antisense morpholino oligonucleotides. This combined approach of sequencing and then knocking down selected genes after validation has identified two novel genes, *trim2a* and *tmem88a* that are required for primitive erythropoiesis and myelopoiesis. Both these cells are initially derived from the posterior blood island (Bertrand et al., 2007) where we have shown by *in situ* hybridisation that both *tmem88a* and *trim2a* are expressed. The exact underlying mechanism is still to be determined but our data suggests that the effect is downstream of *gata1a* because *gata1a* expression by qPCR is normal in both *trim2a* and *tmem88a* morphants.

### 3.1 Trim2a

Trim2a is a member of the TRIM (tripartite motif) family of proteins first identified in a screen of genes up-regulated after induced seizure activity in the hippocampus of mice (Ohkawa et al., 2001). One function of this protein family is to promote ubiquitination of certain proteins via a RING domain. A gene trap screen in mice has recently reported that *Trim2* deficiency causes accumulation of neurofilament light chain and neurodegeneration (Balastik et al., 2008), with no mention of a haematopoietic defect. It is possible that the mouse mutation functions as a hypomorph, because the gene trap integration occurs in intron 6 while the RING domain is found in exon 2 (Balastik et al., 2008); in contrast our splice blocking morpholino induces loss of exon 2.

### 3.2 Tmem88a

The second novel haematopoietic gene identified in this study is *tmem88a*. *TMEM88* was originally identified in a screen for proteins that bind to dishevelled (Lee et al., 2010). This interaction negatively regulates the canonical Wnt signalling pathway, so loss of *tmem88a* should increase Wnt signalling in the zebrafish embryo, and this in turn would be expected to increase numbers of haematopoietic stem cells rather than cause the observed phenotype (Staal and Clevers, 2005). Future work will investigate the mode of action of *tmem88a*.

### 3.3 Conclusion

We have used high-throughput sequencing to catalogue the transcriptome of an enriched population of blood and vascular endothelial cells in the developing zebrafish embryo, and verified our approach by showing that two novel genes thus identified, *trim2a* and *tmem88a*, are required for primitive erythropoiesis and myelopoiesis. This provides a valuable resource in efforts to understand endothelial cell development and haematopoiesis in vertebrate embryos.

## 4 Methods

### 4.1 Zebrafish maintenance, dissociation and fluorescence-activated cell sorting (FACS)

Zebrafish were maintained under standard conditions (Nusslein-Volhard and Dahm, 2002) and staged according to Kimmel et al. (1995). All procedures complied with the UK Home Office requirements. The embryos obtained from in-crossing transgenic Tg(*fli1a:egfp)*^y1^ zebrafish were grown to 26–28 h post fertilisation (hpf) prior to dechorionating with pronase. They were then washed in calcium free Ringer’s solution for 15 min during which time the yolks were removed by gently pipetting up and down. The embryos were transferred to a 50 mm petri dish (Sterilin) and incubated at 28.5 °C in 1× PBS (pH 8), 0.25% trypsin and 1 mM EDTA (Invitrogen) until the embryos were a single cell suspension (approximately 30–40 min). To aid dissociation the solution was agitated by pipetting up and down every 10 min. The digest was stopped by adding CaCl_2_ to a final concentration of 2 mM and foetal calf serum (FCS) to 10%. The cells were centrifuged at 400*g* for 5 min and washed once in PBS before re-suspending in Leibovitz’s L15 medium without phenol red (Invitrogen), 1% FCS and 0.8 mM CaCl_2_. Single cell suspensions were sorted at room temperature using the 488 nm laser on a Cytomation MoFlow high performance cell sorter (Dako). The separated cells were collected in Leibovitz’s L15 medium without phenol red, 20% FBS and 0.8 mM CaCl_2_. The sorted cells were centrifuged at 400*g* for 5 min before re-suspending in 1 ml Trizol (Invitrogen) and storing at −80 °C until RNA extraction was performed according to the manufacturer’s protocol. The maximum time from starting the dissociation until the cells were re-suspended in Trizol was 2 h.

### 4.2 Gene knockdown by morpholino oligonucleotide injection

One-cell Tg(*fli1a:egfp)*^y1^ embryos were injected with 1 nl of custom (translation- or splice-blocking) morpholino oligonucleotide plus zebrafish p53 morpholino, or standard control morpholino (5 or 10 μg/μl each, Gene Tools, USA). See Supplementary Table 3 for sequences. The embryos were inspected under brightfield and epifluorescence microscopy at 24 and 48 hpf for defects in morphology and the development of the vascular system, heart or haematopoietic cells. Morphologically abnormal embryos were excluded from analysis. The effectiveness of knockdown for splice-blocking morpholinos was confirmed by RT-PCR. The primer sequences can be found in Supplementary Table 4.

### 4.3 Whole embryo staining for globin and myeloperoxidase expression

O-dianisidine staining was used to study globin expression (Detrich et al., 1995) and peroxidase staining for myeloid cell expression (Le Guyader et al., 2008).

### 4.4 Illumina RNA-Seq library preparation, sequencing and analysis

Illumina RNA-Seq libraries were made with 3 μg of total RNA according to the manufacturer’s protocol. The only deviation from the protocol was to use the E-gel clone well system (Invitrogen) for fragment size selection. There were two biological replicates for both groups (*gfp+* and *gfp−* sorted cells). 36 base pair single end sequencing was undertaken using an Illumina GA IIx DNA sequencer and the reads were mapped to the Zv8 zebrafish genome and visualised on the UCSC genome browser (http://genome.ucsc.edu/). (Fujita et al., 2011) Fragments per kilobase of transcript per million mapped reads (FKPM) and differential expression levels between experimental groups were determined using Cufflinks (Trapnell et al., 2010). Fold changes were calculated from the FKPM values. For genes where there were no reads in the GFP negative library a value was added to both the GFP positive and negative FKPM values prior to calculating the fold change. This value was calculated using the following formula: 1 + 2√(average GFP positive FKPM value + GFP negative FKPM value). The predicted molecular and biological functions of genes expressed at threefold greater levels in GFP positive cells compared with GFP negative cells in both biological replicates were determined using the Panther Ontology (Thomas et al., 2003). Sequencing results were validated by quantitative RT-PCR (qRT-PCR) using the remaining total RNA used to make the libraries and by whole mount *in situ* hybridisation. The raw data (fastq files) have been submitted to the Sequence Read Archive (SRA059568).

### 4.5 Quantitative RT-PCR

cDNA was transcribed from 0.5 μg of total RNA using Superscript II (Invitrogen) according to the manufacturer’s instructions and diluted to 50 μl for RT-PCR. Quantitative RT-PCR was performed in duplicate in 10 μl reactions using 2.5 μl of cDNA, 1× Lightcycler Mastermix (Roche) and 0.5 mM forward and reverse specific primers on a Lightcycler LC480 (Roche) according to the manufacturer’s instructions. Primer pairs were designed using NCBI primer-BLAST and are found in Supplementary Table 5. Expression levels were compared to a standard curve and values normalised to *ef1α* and expressed as the fold change relative to the GFP negative group.

### 4.6 Whole mount *in situ* hybridisation

Probes were made by PCR amplifying either the whole open reading frame (ORF, if less than 2 kb) or about 1 kb of the 3′ end of the ORF (if greater 2 kb) of the genes of interest using Sahara mix (Bioline) according to the protocol provided. The primer pairs used for each gene are in Supplementary Table 6. PCR-amplified transcripts were TOPO cloned (Invitrogen) and Sanger sequenced to determine the orientation of the transcript. Sense and anti-sense *in situ* probes were made using the appropriate DIG RNA labelling kit (Roche). Whole mount *in situ* hybridisation was performed as described (Nusslein-Volhard and Dahm, 2002).

### 4.7 Imaging

A Leica APO dissecting microscope mounted with a Coolpix 4500 camera (Nikon) was used to image and photograph embryos after *in situ* hybridisation and O-dianisidine staining.

## Authorship

JEC, EP & AE designed and performed experiments and analysed data, JEC wrote the paper, CRB analysed data, AS performed the high-throughput sequencing, NWM and JCS designed experiments, analysed data and wrote the paper.

## Figures and Tables

**Table 1:** Biological function enrichment for the 754 genes identified in the *gfp+* population of cells. Biological functions were assigned using the Panther Ontology and fold changes determined compared to the expected number of genes with a particular function in the genome.

Panther biological function	Fold change	*p*-Value
Neuronal activities	−2.28	8.57E−06
Immunity and defence	1.45	5.27E−05
Transport	1.42	1.91E−04
Nucleoside, nucleotide and nucleic acid metabolism	−1.31	2.42E−04
Blood circulation and gas exchange	2.77	5.14E−04
Intracellular protein traffic	1.4	1.82E−03
Homeostasis	1.85	3.49E−03
Lipid, fatty acid and steroid metabolism	1.39	6.96E−03
Carbohydrate metabolism	1.46	7.08E−03
Signal transduction	1.14	0.014
Protein metabolism and modification	1.16	0.017
Cell cycle	−1.4	0.018
Developmental processes	1.18	0.020
Sensory perception	−1.59	0.024
Coenzyme and prosthetic group metabolism	1.74	0.026
Cell proliferation and differentiation	1.24	0.042
Biological process unclassified	1.03	0.091

**Table 2:** Ten genes validated by qRT-PCR and *in situ* hybridisation selected for morpholino knockdown. Fold changes are the means of both biological replicates. ♢ Indicates no reads in the *gfp-* samples, so a factor has been added to each read count prior to calculating a fold change (see Section 4 for details). EC: vascular endothelial cell; RBC: erythrocyte.

Ensembl ID	Gene	Sequencing fold change	qRT-PCR fold change	*In situ* expression pattern
ENSDARG00000061747	*CC058*	14.72♢	46.63	EC, RBC
ENSDARG00000020031	*cldn11a*	14.80	59.79	EC
ENSDARG00000041724	*glipr2*	43.06	65.15	EC, RBC
ENSDARG00000037883	*prcp*	6.15	18.93	EC, RBC
ENSDARG00000063370	*sgk2a*	13.41	11.56	EC, RBC
ENSDARG00000059682	*slc43a3*	33.85	52.83	EC, RBC
ENSDARG00000043604	*tmem205*	18.47	53.79	EC, RBC
ENSDARG00000056920	*tmem88a*	10.09	28.76	EC, RBC
ENSDARG00000031817	*trim2a*	6.37	7.34	EC, RBC
ENSDARG00000034453	*unc119a*	15.42	61.76	EC, RBC

## Appendix A Supplementary data

Supplementary Table 1 Summary of massively parallel sequencing and expression data for genes enriched at least three-fold in GFP positive cells.

Supplementary Tables 2–5 This document contains Supplementary Tables 1–5.
