# Supplementary material for: Global analysis of the haematopoietic and endothelial transcriptome during zebrafish development
Source: Mech Dev. 2013 Feb;130(2-3):122–31. doi: 10.1016/j.mod.2012.10.002 (PMC3580284; doi:10.1016/j.mod.2012.10.002)
Supplement: Supplementary Tables 2–5 — This document contains Supplementary Tables 1–5. [file mmc2.docx]

**Supplementary Table 1.** **Summary of massively parallel sequencing and expression data for genes enriched at least three-fold in GFP positive cells.** Ensembl ID and FKPM values were generated by Cufflinks (Trapnell et al., 2010). Expression patterns were determined using data from ZFIN and Pubmed. ec: vascular endothelial cell; nc: neural crest; pa: pharyngeal arch; plt: platelet; pnd: pronephric duct; rbc: red blood cell; wbc: white blood cell. See Excel file named supplementary Table 1.**Supplementary Table 2. 85 genes selected for validation and further evaluation.** Fold changes are the mean of both biological replicates. ◊ indicates no reads in the *gfp-* samples and therefore a factor has been added to each read count prior to calculating a fold change (see Methods for details). EC: vascular endothelial cell; RBC: erythrocyte; WBC: myeloid cell; PND: pronephric duct; TB: tailbud.

| **Ensembl ID** | **Gene** | **Sequencing fold change** | **qRT-PCR fold change** | ***In situ* expression pattern** |
| --- | --- | --- | --- | --- |
| ENSDARG00000056200 | *abcb9* | 34.26 | 21.74 |  |
| ENSDARG00000077782 | *acer2* | 20.90 | 43.59 | EC, RBC |
| ENSDARG00000057273 | *alox5* | 7.17 | 7.15 | PND |
| ENSDARG00000026787 | *aqp7* | 4.92 | 10.06 | EC |
| ENSDARG00000045141 | *aqp8a.1* | 23.40 | 60.49 | EC |
| ENSDARG00000022934 | *arl4a* | 11.47 | 39.68 | EC, RBC |
| ENSDARG00000061747 | *CC058* | 14.72 ◊ | 46.63 | EC, RBC |
| ENSDARG00000020031 | *cldn11a* | 14.80 | 59.79 | EC |
| ENSDARG00000060893 | *collagen alpha-2(VIII)* | 6.31 | 20.43 |  |
| ENSDARG00000031426 | *csrnp1a* | 18.82 | Poor primers |  |
| ENSDARG00000076789 | *cx32.2* | 13.84 ◊ | 46.31 | Non specific |
| ENSDARG00000018283 | *cyba* | 15.80 | 28.17 |  |
| ENSDARG00000039932 | *cyth4b* | 5.93 ◊ | 54.73 |  |
| ENSDARG00000013153 | *denn3b* | 20.00 | 22.18 | Non-specific |
| ENSDARG00000040930 | *deptor* | 9.89 | 155.66 |  |
| ENSDARG00000018688 | *elk3* | 3.90 | 13.03 | EC |
| ENSDARG00000074998 | novel protein | 15.97 | 197.07 |  |
| ENSDARG00000076346 | novel protein | 44.91 | 23.37 |  |
| ENSDARG00000055398 | *foxc1b* | 7.33 | 25.68 |  |
| ENSDARG00000051853 | *galns* | 6.95 | 7.00 |  |
| ENSDARG00000041724 | *glipr2* | 43.06 | 65.15 | EC, RBC |
| ENSDARG00000057619 | *gpr141* | 9.97 ◊ | 5.93 |  |
| ENSDARG00000070404 | *inka1b* | 9.97 | 5.93 | PA, EC, TB |
| ENSDARG00000044318 | *integrin beta 7* | 8.84 | 21.71 |  |
| ENSDARG00000060396 | *lamc3* | 7.37 | 4.88 | EC |
| ENSDARG00000003022 | *limk1* | 8.74 | 2.82 |  |
| ENSDARG00000070792 | *lrrc15* | 17.02 ◊ | 7246.51 | RBC |
| ENSDARG00000038681 | *map4l* | 3.12 ◊ | 2.48 |  |
| ENSDARG00000052978 | *mbnl1* | 42.80 | 36.63 | RBC |
| ENSDARG00000071413 | *mier1* | 6.95 | 6.31 |  |
| ENSDARG00000031855 | *mst1* | 13.04 | 23.31 |  |
| ENSDARG00000009782 | *myh11a* | 44.71 | 71.54 |  |
| ENSDARG00000057206 | *nmt1* | 76.68 | 57.87 | RBC, WBC |
| ENSDARG00000074175 | *Novel Zn finger* | 9.54 | 7.77 |  |
| ENSDARG00000009390 | *npl* | 4.68 | 9.99 |  |
| ENSDARG00000069031 | *plac8* | 14.95 ◊ | 197.59 | EC, RBC |
| ENSDARG00000015278 | *plxnc1* | 13.10 | 10.95 | Non specific |
| ENSDARG00000037883 | *prcp* | 6.15 | 18.93 | EC, RBC |
| ENSDARG00000012340 | *ptpn11b* | 14.29 ◊ | 21.26 |  |
| ENSDARG00000079291 | *rapgef3* | 7.16 | 1.12 |  |
| ENSDARG00000076768 | *reps2* | 10.69 | 9.35 | EC, RBC |
| ENSDARG00000058725 | *rfesd* | 72.04 | 158.95 |  |
| ENSDARG00000007727 | *rgl2* | 10.45 | Poor primers |  |
| ENSDARG00000058348 | *scinlb* | 8.45 | 6.23 |  |
| ENSDARG00000063370 | *sgk2a* | 13.41 | 11.56 | EC, RBC |
| ENSDARG00000078547 | *si:ch211-264f5.2* | 19.71 | 79.00 |  |
| ENSDARG00000071052 | *si:dkey-150i13.2* | 53.87 | 29.40 |  |
| ENSDARG00000058160 | *si:dkey-196h17.3* | 15.05 | 41.82 |  |
| ENSDARG00000078734 | *si:dkey-240a9.5* | 27.41 | 27.97 |  |
| ENSDARG00000040741 | *si:dkeyp-94h10.1* | 15.87 | 54.05 |  |
| ENSDARG00000058731 | *slc2a6* | 33.13 | 25.37 |  |
| ENSDARG00000054447 | *slc29a1* | 31.46 ◊ | 38.84 | EC, RBC |
| ENSDARG00000009901 | *slc38a3* | 25.04 ◊ | 71.97 | RBC |
| ENSDARG00000059682 | *slc43a3* | 33.85 | 52.83 | EC, RBC |
| ENSDARG00000004405 | *snx10a* | 3.99 ◊ | 4.57 | Non specific |
| ENSDARG00000040474 | *snx10b* | 4.32 ◊ | 6.08 |  |
| ENSDARG00000008678 | *snx3* | 8.49 | 9.16 | EC, RBC |
| ENSDARG00000074842 | *snx8* | 3.17 | 12.88 | RBC |
| ENSDARG00000000837 | *snx9* | 24.68 | 56.60 | RBC |
| ENSDARG00000041006 | *st3gal4* | 25.08 | 17.99 | RBC |
| ENSDARG00000033170 | *sult2st1* | 3.12 | 7.57 | Non specific |
| ENSDARG00000058839 | *susd1* | 81.99 | 80.10 |  |
| ENSDARG00000034895 | *tgfb1b* | 15.36 | 62.00 | EC, RBC |
| ENSDARG00000074322 | *tiarin* | 16.19 ◊ | 87.34 |  |
| ENSDARG00000060668 | *tm4sf18* | 7.10 | 7.01 |  |
| ENSDARG00000043604 | *tmem205* | 18.47 | 53.79 | EC, RBC |
| ENSDARG00000056920 | *tmem88a* | 10.09 | 28.76 | EC, RBC |
| ENSDARG00000056985 | *tpte* | 3.94 | 7.58 |  |
| ENSDARG00000031817 | *trim2a* | 6.37 | 7.34 | EC, RBC |
| ENSDARG00000034453 | *unc119a* | 15.42 | 61.76 | EC, RBC |
| ENSDARG00000054363 | *wu:fd14a06* | 21.41 | 54.50 |  |
| ENSDARG00000054099 | *zgc:100914* | 5.67 | 6.28 | EC, RBC |
| ENSDARG00000075136 | *zgc:101030* | 9.75 | 3.36 | RBC |
| ENSDARG00000037852 | *zgc:101663* | 24.83 | 18.45 |  |
| ENSDARG00000033285 | *zgc:101897* | 9.65 | 21.97 |  |
| ENSDARG00000020979 | *zgc:113070* | 63.11 | 54.32 |  |
| ENSDARG00000059857 | *zgc:153046* | 15.97 | 44.42 |  |
| ENSDARG00000056453 | zgc:153096 | 3.36 | 14.63 |  |
| ENSDARG00000007769 | *zgc:153723* | 21.81 | 80.16 |  |
| ENSDARG00000061551 | *zgc:153898* | 29.43 | 13.77 | Non specific |
| ENSDARG00000063345 | *zgc:158288* | 16.90 | 57.82 | RBC |
| ENSDARG00000019651 | *zgc:162239* | 9.80 | 11.66 |  |
| ENSDARG00000058100 | *zgc:171538* | 30.77 | 22.95 | RBC |
| ENSDARG00000076189 | *zgc:175202* | 22.86 | 47.22 |  |
| ENSDARG00000006508 | *zgc:92316* | 22.28 | 28.26 | Non specific |

**Supplementary Table 3. Antisense morpholino oligonucleotides used in this study and their mechanisms of action.** AUG = translation blocking, Spl = splice blocking morpholino with splice junction targeted by morpholino in brackets.

| **Gene** | **Morpholino sequence** | **Morpholino type** |
| --- | --- | --- |
| *cc058* | ACCTGCAGTGAAATAAACAGGGTCT | Spl (i1e2) |
| *clnd11a* | TTGTTTTTTTTCAGACGTACCTGGA | Spl (e1i1) |
| *glipr2* | CTCAAAACTGCTGCCCGCCATGCAT | ATG |
| *prcp* | GAAATTAGCAATGACACTTACCTGC | Spl (e1i1) |
| *sgk2a* | GGGTTGGATCGTAATAAGCCATCTT | ATG |
| *slc43a3* | ACCGCAGTCTAAAGAGAAAAGCAGA | Spl (i1e2) |
| *tmem205* | CAGTCGGATCTCCCTCAGTAGCCAT | ATG |
| *tmem88a* | GCATTCTCACTCCACACATACCGTT | Spl (e2i2) |
| *tmem88a* | TCGTGGAAGACTCATCTTGCCGTTC | ATG |
| *trim2a* | ACCATTAAAAACACTACCTTTCACA | Spl (e2i2) |
| *trim2a* | GGCCATCCTAACACTCTATCAAATA | ATG |
| *unc119a* | ATTGACATTACTGAACTTACTGTCT | Spl (e1i1) |

**Supplementary Table 4. Primer sequences used to confirm effectiveness of morpholino knockdown.**

| **Gene** | **Forward primer** | **Reverse primer** |
| --- | --- | --- |
| *cc058* | AGACCCATCACCAGCACCCCAA | GCCCGCTGTGCTGTCTGTGT |
| *slc43a3* | ACCTGTGCAGCAACGCGACA | GCCGCCCACAGCTATGAAGGA |
| *tmem88a* | GCCAGTCTGAGGAATCTGCCCA | CAGTCTGCTCAAACCCACACGC |
| *trim2a* | GTGGCTCACGTTTCATCAAGAC | TTTCCTCCATGATTGGGACAGG |
| *cldn11a* | GGCTAATCGTCGCCACTGCCA | GCACCATCACAACGGCAGGCAA |
| *prcp* | AAAGCGCTGGCTCTCAAACCTCG | GCAAAGCTCCCAGCTCCTCTGC |
| *unc119a* | AGTGAAGCAAGGCTGCGACG | GGAAGGCCGGGGTGAACTGG |

**Supplementary Table 5. Primer sequences used for qRT-PCR validation.**

| **Ensembl ID** | **Gene** | **Forward sequence** | **Reverse sequence** |
| --- | --- | --- | --- |
| ENSDARG00000056200 | *abcb9* | CCAAGGTGGCACTGGTGGCTC | TGCCCTTCTCTCCGACCCCA |
| ENSDARG00000077782 | *acer2* | TCTGTGGTGGACGCTAGCGCT | GCACCCCGATGAAAGCCCAC |
| ENSDARG00000057273 | *alox5* | CCTGACCGAGGACGCTCC | GAGAGTGGTGAATTACACAGCCA |
| ENSDARG00000026787 | *aqp7* | AATCCCACACGGGACCTGGGG | ACTGCTGGCCGTGTTGTAGTGTT |
| ENSDARG00000045141 | *aqp8a.1* | CTGCATTGGACTCACTGTGACGGC | ACAGTGACCAGAGCGCCGGTA |
| ENSDARG00000022934 | *arl4a* | CGCCCGCTCCACTGAAAATCAA | TGATGGCGCAAGTGGGCTGC |
| ENSDARG00000061747 | *CC058* | CACAGACAGCACAGCGGGCT | GGAAGCGTGAGGTGGCCCTG |
| ENSDARG00000020031 | *cldn11a* | AGGGGTTGTGGGCAGACTGTGT | CCTTCTCGTGATGGGCCCCGA |
| ENSDARG00000060893 | *collagen alpha-2(VIII)* | CCCCTGGCCCCTCTAACGGT | TGCCTGTGGCAGGGTTGTAAGC |
| ENSDARG00000031426 | *csrnp1a* | ACGCGGGCTGGTATTGGTGT | CTCAATGCGGCCTGCGGGAT |
| ENSDARG00000076789 | *cx32.2* | GAGGTGGCCTTCATCGTCGGAC | GAGACACGCAAGCCACCACCA |
| ENSDARG00000018283 | *cyba* | GCAAGCTTTGGCTGCTGGACTC | ACTGGTGCCTTTGCCCCGTTT |
| ENSDARG00000039932 | *cyth4b* | GACAGACGGACGCGTGGTGG | GCGCGCGCTCTCGTATCTCA |
| ENSDARG00000013153 | *denn3b* | AGAGTGTCTGCGCTGCCTCCA | AGCCGAGACCACACCACAGCT |
| ENSDARG00000040930 | *deptor* | AGGCGGCGGCTGATAGAGCT | GGGCTGCTGCTGAGTGTGGG |
| ENSDARG00000004232 | *dlb* | GTTTCACTCATTTCTCCGGG | TCATTAAACACGGAATCGCA |
| ENSDARG00000020850 | ef1α | GAGAACCTCTGCCACTCAGG | CTCAAGGTGAAACGTCCCAT |
| ENSDARG00000018688 | *elk3* | CGAAAGCCCGGAAACCCAAAGG | GTAGTCTGGCCGGGCTGAGG |
| ENSDARG00000074998 | *ENSDARG00000074998* | GCTCAGATTCCACTCAGCACAGT | ACCGTGGCCTCAGGAGTGG |
| ENSDARG00000076346 | *ENSDARG00000076346* | CGAGACGAAGGATCTTTCAGCTGC | ACTTCAGCGTTCCTTTATCTGCGAA |
| ENSDARG00000054632 | *fli1a* | AGCGCTACGCCTACAAGTTC | AGCTCCAGTATGGGGTTGTG |
| ENSDARG00000015717 | *flt4* | TGGTTTTGGCATCAGATGAA | GGAGAAGTAGTCGCTGACGC |
| ENSDARG00000055398 | *foxc1b* | CAGTTCCGCATCGCCGCTTCT | TGAACTGGCACCGGGATTGCA |
| ENSDARG00000051853 | *galns* | ACCCATGCACCTGTCTACGCCT | AGGCGATGGCTGGTTCCCTCA |
| ENSDARG00000041724 | *glipr2* | TGCACAGCAACGGAGACCATGG | CCTCCTGGAAGGTACTGTCCCACC |
| ENSDARG00000057619 | *gpr141* | GCATTGGGCATGAGCGCTTTTG | GGACGCAGGCCTGAACGCAC |
| ENSDARG00000070404 | *inka1b* | GTCGTCTGCATGCGTTCGCA | TGCCTGCGATTTTCCGTCGCT |
| ENSDARG00000044318 | *integrin beta 7* | GTGCGAGTGCTCACCTAGCCA | TTGAACAGCTCGCAAGCGTCCA |
| ENSDARG00000015815 | *kdrl* | GCCCAGAGAGTGTGAAGACC | CCTCCAGCAGAACTGACTCC |
| ENSDARG00000060396 | *lamc3* | CTAAACAGGCCGAGGCGTCG | TGTCTCCTCTGCTTCCAATGTTGCC |
| ENSDARG00000003022 | *limk1* | CAGACTTCGGGCTGTCGCGG | GCCCGCGGCAGATAATCCGG |
| ENSDARG00000070792 | *lrrc15* | CCTCGTGATGTGCTCTTCTCCTCCA | AGACCTTGACCCGCAGCATCAC |
| ENSDARG00000038681 | *map4l* | TGGAGGAGCAGGCGACTCAA | TGCTCTTCCAGCCGTACCAAACA |
| ENSDARG00000052978 | *mbnl1* | CCTGGCCTGATGTCCGCAGA | GGTCGATCATTGTGCTGTCGGCA |
| ENSDARG00000071413 | *mier1* | AACGGGAGCGACAGTGGACCT | TCACCCACTGAGCGTGTGCG |
| ENSDARG00000031855 | *mst1* | TGCCTTCCTCCCGAGCGCTA | CGCACGCGGCCCTTGAAGTA |
| ENSDARG00000009782 | *myh11a* | ACAGGCTGCATTGGCGCGA | CTCATGGACGCGGCTCTCGT |
| ENSDARG00000030110 | *myod* | GGGCCCAACGTGTCAGACGA | GTTGAGGGCAGCTGGTCGGG |
| ENSDARG00000057206 | *nmt1* | CATTGCAGCGGGCTCTCAAGCT | CCAGTGCTCAACATCCTCCAGGGT |
| ENSDARG00000074175 | *Novel Zn finger* | TCGTGCGCTCAGTGTGGACAC | TTTACGGCAGCGTGGACTTTCC |
| ENSDARG00000009390 | *npl* | ATAACGGCAACCACACGCAGACC | TGGAAGTCGAGGAGGACCCATTGG |
| ENSDARG00000069031 | *plac8* | GCGGTGATGCCAGCACATTGC | GTCCTCACACACATTGCCCCGG |
| ENSDARG00000015278 | *plxnc1* | TCCCAAACTTCTCCTGCGCCG | GGGGCTCAAAGTCCTGCGCTT |
| ENSDARG00000037883 | *prcp* | GCCTCAGCCATGGAGCTTCCA | AGCGCAGATCAAGATGGTGAGCAC |
| ENSDARG00000012340 | *ptpn11b* | TGTGTGCGGTACTGGCCAGAC | GCGCTCTGGGTCCGGTTCAC |
| ENSDARG00000079291 | *rapgef3* | AGACGGACGGTGTTTGCCTCG | CCACTCTGCGCTCGTCCCTTT |
| ENSDARG00000076768 | *reps2* | GCCAATGCAGCGAGTCAGCG | GTCGAGTGAGGAGGCGCGTG |
| ENSDARG00000058725 | *rfesd* | GCGACGCAGAGGCAGATGTCAAT | TGAAAGTCAAGCCGCTGCAGTGA |
| ENSDARG00000007727 | *rgl2* | AGGACTCGGCTCACCTGGTGC | AGGACTCGGCTCACCTGGTGC |
| ENSDARG00000058348 | *scinlb* | AGTCATTCTGGGCGGCTTTGGG | CGGCCAGCGGGATCAGACTCC |
| ENSDARG00000063370 | *sgk2a* | ACCACCTTCTGCGGCACTCC | CGACTCGGACTTTCCCGGCG |
| ENSDARG00000078547 | *si:ch211-264f5.2* | ACGCGGGCTGGTATTGGTGT | TGCTGCTTTGGGACGAGTCCTCT |
| ENSDARG00000071052 | *si:dkey-150i13.2* | GCAGGCATGGTGGCCCAGTAA | TCGTGGTCTCCCGAGTTCGTTC |
| ENSDARG00000058160 | *si:dkey-196h17.3* | GAGCAAGCAGCCCAACACTGA | GCCCACTTTCCTGTACAAGTCCTTC |
| ENSDARG00000078734 | *si:dkey-240a9.5* | TGGGGCAGCGCAGTTTCACC | ATCGTGGGCCCTGTGGTCGT |
| ENSDARG00000040741 | *si:dkeyp-94h10.1* | CCATCGCTTGGTGCCTCCGT | ACACTGCCGACACACGAGGG |
| ENSDARG00000058731 | *slc2a6* | ACCATGTACACCCACCAAACACC | GACACCACGCGCACCCAAAG |
| ENSDARG00000054447 | *slc29a1* | TCCCTCGCCATCGCTTCATATGT | GTCCACAGTGACCGCAGGAAACG |
| ENSDARG00000009901 | *slc38a3* | TGTGCCTCCTGTTTGCCGTCAA | TGACCGCCCACCGACTGCTT |
| ENSDARG00000059682 | *slc43a3* | GTCAGTCATGCAGCTCCGCCA | GCGCAGGTCTGCTTCCCGTT |
| ENSDARG00000004405 | *snx10a* | CGGCGAAGCACGTTTCCCTGT | CCTCGGGGTTTGTGGCTTGAAA |
| ENSDARG00000040474 | *snx10b* | AAGGCAGGACTCGCTACACTGT | TAACTGGCACGTTGGGTAGGC |
| ENSDARG00000008678 | *snx3* | GAATGACGCGTACGGGCCAC | ATCCCCACGGAAAGGAAGCTGC |
| ENSDARG00000074842 | *snx8* | GCCCATCCTGGTGTCTGGCAG | CGCTCGCTGGTGATCGTGCAA |
| ENSDARG00000000837 | *snx9* | ACCATGCAGCGGAGTGGAGC | CTGGACCAACCTCGCCTGGA |
| ENSDARG00000041006 | *st3gal4* | AGGATCTGCGCTGGCTCAAAGAGA | TTGTTTGCGCGGCTGCACGG |
| ENSDARG00000033170 | *sult2st1* | GGTGCCGGAGGAGTTCATGGAC | GGTGTTCAGTCCCATGGAAAGCTG |
| ENSDARG00000058839 | *susd1* | ACTGTGGCGTCCCCGTCTCTC | GGCCCACAGTTGATCTCTTCGCA |
| ENSDARG00000034895 | *tgfb1b* | GGCTGGCAAGTGGGTGTCGT | TGGTCCGCTAAGAGGCCCGT |
| ENSDARG00000074322 | *tiarin* | CCACCCCCTACACTTTCCCCAGG | TGTTCTCGTCTCCTGGAGCGGG |
| ENSDARG00000060668 | *tm4sf18* | CAGGAGCAGCACGTGACCCAAT | GCCTCTCAGTAGCGCCAGACC |
| ENSDARG00000043604 | *tmem205* | GGGGAATGCAGGTGTGGGTGTC | TGCCTTCATGCCAGTCGAGCA |
| ENSDARG00000056920 | *tmem88a* | CCTGCCATCGCTCGTCATGGT | AGACGGCACGGCTGTATGGGA |
| ENSDARG00000056985 | *tpte* | AGTGCTGGTCTGCCGAAGGGT | GGCTCAGTGAAAGACAGCGTCA |
| ENSDARG00000031817 | *trim2a* | GCTCCAACGGGTGTGGCAGT | GCACAAGGGCGGCAGCGTTA |
| ENSDARG00000034453 | *unc119a* | TGAGACTGGGGAGGACAGAATGG | TCAGACAGCGGTGGGAACTCGT |
| ENSDARG00000054363 | *wu:fd14a06* | GACGTTCCTGCGGCATTCCCA | GTGCAGCTCGCCTCCTGATACG |
| ENSDARG00000054099 | *zgc:100914* | GGGTGGAACCATGCGGCTGG | CCGCTCCCCCTCTAGGTCCTG |
| ENSDARG00000075136 | *zgc:101030* | GCGCAGGAATCAGCAGAAGAGCT | CCCTTGAGTCGCACAGGCCG |
| ENSDARG00000037852 | *zgc:101663* | ACGGTGGGCTTGTCGTCGGT | CGTGGATGACCAGCAGACGGC |
| ENSDARG00000033285 | *zgc:101897* | CGACCACTACAATGGCTTCATCTCC | CCTCGTCCAGGTACTCGCAGGT |
| ENSDARG00000020979 | *zgc:113070* | CTCGTCCTGCCTCAACCGGG | CACGTGCAGCGTTCCTCAGGT |
| ENSDARG00000059857 | *zgc:153046* | AGCTGCACTTCACCGGTGGT | GACCGGCTCACGCTGACCTG |
| ENSDARG00000056453 | zgc:153096 | GTCACTGCGCTCCGAACGGA | CTTTTCCTGAGGCGCCCCCA |
| ENSDARG00000007769 | *zgc:153723* | TCCCCAAGGAGATCATCGACCAC | GGCACTAGAGTCTCCCCGGGC |
| ENSDARG00000061551 | *zgc:153898* | ACGCAGATGGACAGGAGTCACT | CCCGGTTCACCCTGATGCCTG |
| ENSDARG00000063345 | *zgc:158288* | ACGCAGGTCTGGGTGCTCGA | CTGAATCACCTCATCAGTGGCTCTG |
| ENSDARG00000019651 | *zgc:162239* | CAAAGATGAGGTCGGGGTGAACC | TCATCCGGGTCACACTCTGGGAC |
| ENSDARG00000058100 | *zgc:171538* | TGGCGTACGAACTGCTGGTCT | TGCAAAATGTCTTCACACACCCTGT |
| ENSDARG00000076189 | *zgc:175202* | AACCACGTGCGGCTCAGCAG | CATTGCGGGCAGAGCAGGCT |
| ENSDARG00000006508 | *zgc:92316* | AGTCACACCCTCTCCCCGCC | CGTCGAGCACGGAGTCGTCA |

**Supplementary Table 6. Primer sequences used to amplify genes for *in situ* probes.** PL: partial length; FL: full-length open reading frame.

| **Gene** | **Forward sequence** | **Reverse sequence** | **FL or PL** |
| --- | --- | --- | --- |
| acer2 | TGATGGACGCGCATCTGTGGG | TCAGGTCACCTTGATGGAGGGTTTTCG | FL |
| alox5 | AACACCAAGGCTCGTGAGCAGC | GCACTTCTGGTTTGATGAGAGTGGT | PL |
| aqp7 | GAAACAGGAAGTCAGCATAATGGAAGATGG | TGGGAACAGGACTCACACAGGT | FL |
| aqp8a.1 | GCTCCGGGCAGAAATCAAAAATGACC | GCTTGCAATCCTCTTCAGTTCCTTCTTCC | FL |
| arl4a | GGGTCTGGACTCTGCAGGCAA | GCTTGCTGGCTCTATAACCAACTCTAG | PL |
| CC058 | CTCTGGGGTTTATGATGCAGTGATGG | CTAGAACTTGTCACTGTAGAGACATTCCG | FL |
| cldn11a | TGCGCACACTGTACACCATGGCC | GGTCCTTCACCAGCGATTGTCCC | FL |
| cx 32.2 | AGTCCTGACCAACTGAGCGGCC | CGTGACCCTTTAAGCCTCAGGTTTGG | FL |
| Denn3b | TCAGGTGTGGATTGGCTCGGAGG | ACTTGCATGCTTCAAATCTTGACGC | PL |
| elk3 | GAACTACGACAAACTTAGCCGAGCG | GTAGTCTGGCCGGGCTGAGGG | PL |
| glipr2 | CCTTCTGGGAGGTGACCTGCTGAA | TGTGCATGTGTGTGGCAGGGGA | PL |
| inka1b | CAAACATGCTGTGTGTTCGCAATTCAG | ATCCCGTCCATCTCACGCATCAGA | FL |
| lamc3 | AACACAACGCAGAGATAGCGGACA | ACTCCAGCTGATTCTTGGCCGT | PL |
| lrrc15 | CAACACACTGAGCCTTTAGATGATGCT | CAACACACTGAGCCTTTAGATGATGCT | FL |
| nmt1 | GCTCTTTGTCCTCCTGGGTGGC | TCCGGGCATCTCCAGTTGTAGAGGT | PL |
| plac8 | GACAGCAATGATGGCCTTCAGTC | GACTGAAGGCCATCATTGCTGTC | FL |
| plxnc1 | GCCAAGCGCAGGACTTTGAGC | GGAGAGCCGCCGATTCATTGA | PL |
| prcp | CTCAAGATGACGACCAGTGACTGAAGTC | TTATGTAGTTGCTGCATGTTTGATCCACT | FL |
| Reps2 | CTCACGCGCCTCCTCACTCG | ACCATTTGGCTGGAAGTGCAGTGC | PL |
| sgk2a | CACAAGATGGCTTATTACGATCCAACCC | TGTGTGTGTGTCCAGAAACCTCCA | FL |
| slc29a1 | ACCGGCTTCATACACGACACCC | GGATAGAGACGCTCCGAGGGCC | PL |
| slc38a3 | ACCTGCTGACTGCTATCTTTGGCT | TGGACCCCAAAACATGCACTATGAACA | PL |
| slc43a3 | CCTTCATAGCTGTGGGCGGC | TTGTTGAGGGCGAAGCAGGGA | PL |
| snx10a | TTAATCACGTCAGCGCGAGGAGC | GGTTCTAAGCAGCAATTGGGTCTCTTTT | PL |
| snx3 | TGCTCAAACCTGTAACGGGAAGC | TGGACGCCCTATGCTTCATGC | PL |
| snx8 | GGACGTGCCATCGCGCTCAC | CCATCCCCAAAAAGACTTTTCAGCTTG | PL |
| snx9 | GATACCACGATGGCAGTGAAGGC | GACTGTGTCCTCATATGGTGGTGTACTG | FL |
| st3gal4 | GCGATGTTGTGCACGTGGCAG | TGTGATGTGTGTGTGCCGCTTGG | PL |
| sult2st1 | GACAGAAAATGACGGAAGCAGAGCTG | TCGAGCACCAAAGGCTACAGTGA | FL |
| tgfb1b | CTCAGTGTACAACAGCACCATGGAGCTC | GGCAGTGGGTCTAGAACTTGGAGGTAC | FL |
| tmem205 | TGGGCATGAGCTCCAACAGGGA | TTGACACGTCTAAAACTGGTCCACAAA | PL |
| tmem88a | ATGAACGGCAAGATGAGTCTTCCACG | TGCTCAAACCCACACGCTGCC | FL |
| trim2a | ACCAATCTCCAGGGAGTTGCTGCT | GCACAAGGGCGGCAGCGTTA | PL |
| unc119a | CGCGTTCAGAGATGAAAGTGAAGC | CTACGGTCCTCCATTGTAAGAATAATCGG | FL |
| zgc:100914 | CTGGAGGATCAGGGCATGGTTCAC | GGTGAGGGTTTGATCGGGCGAG | PL |
| zgc:101030 | ACTTTCTCTGGTGTTTGAGCGGTTT | ACAGCGTCGCTCATCAAGTCCT | FL |
| zgc:153898 | GGGGCTCCGTTTGCTCCGTTT | CCCAGTCCCACCGTAGCCTGT | PL |
| zgc:158288 | CGCTGCTCTCCGTGATTCTGGC | GTGGTGTCAGAGCTTTATTTCACTGCACA | PL |
| zgc:171538 | GGTGCAAAGATGAAAAAGAAAACAGTGG | CCCTTTGCAAAATGTCTTCACACACCC | FL |
| zgc:92316 | CAGTCACACCCTCTCCCCGCC | ACGCACTGCTGATGCCAGTCT | PL |
